# Time Course of Performance Indexes, Oxidative Stress, Inflammation, and Muscle Damage Markers after a Female Futsal Match

**DOI:** 10.3390/sports11070127

**Published:** 2023-06-29

**Authors:** Athanasios Souglis, Dimitrios I. Bourdas, Aristotelis Gioldasis, Ioannis Ispirlidis, Anastassios Philippou, Emmanouil Zacharakis, Alexandros Apostolidis, Georgios Efthymiou, Antonios K. Travlos

**Affiliations:** 1 Section of Didactics and Coaching in Sport Games, School of Physical Education & Sport Science, National and Kapodistrian University of Athens, 41 Ethnikis Antistasis, 17237 Daphne, Greece; asouglis@phed.uoa.gr (A.S.); giold_telis@yahoo.gr (A.G.); emzach@phed.uoa.gr (E.Z.); alexapo@phed.uoa.gr (A.A.); 2 Section of Sport Medicine & Biology of Exercise, School of Physical Education and Sports Science, National and Kapodistrian University of Athens, 41 Ethnikis Antistasis, 17237 Daphne, Greece; dbourdas@phed.uoa.gr; 3 School of Physical Education & Sport Science, Democritus University of Thrace, Panepistimioupoli, 69100 Komotini, Greece; iispyrli@phyed.duth.gr; 4 Department of Physiology, Medical School, National and Kapodistrian University of Athens, 75 Mikras Asias, 11527 Athens, Greece; 5 Department of Rheumatology and Clinical Immunology, Faculty of Medicine, School of Health Sciences, University of Thessaly, Papakiriazi 24, 41222 Larissa, Greece; gefthymiou@med.uth.gr; 6 Department of Sports Organization and Management, Faculty of Human Movement and Quality of Life Sciences, University of Peloponnese, Efstathiou and Stamatikis Valioti & Plataion Avenue, 23100 Sparta, Greece; atravlos@uop.gr

**Keywords:** soccer, games, recovery, physical activity, sport, injury, training

## Abstract

Background: Our aims were to investigate the time-course effects of a futsal match on performance, oxidative stress, and muscle damage markers, as well as inflammatory and antioxidant responses during a 6-day post-match period. Methods: Thirty-four female high-level futsal players were assessed on several oxidative stress, inflammation, subjective muscle soreness, subjective rate perceived exertion, and performance tests before a futsal match, immediately after, and 24 h to 144 h after. Results: Counter movement jump, 20 m, and 10 m sprints performance significantly decreased immediately after the match (*p* < 0.05) and returned to baseline 72 h post-match (*p* > 0.05). Delayed onset muscle soreness peaked 24 h post-match and rate perceived exertion peaked post-match (*p* < 0.05) and returned to baseline 96 h post-match (*p* > 0.05). Inflammatory biomarkers peaked at 24 h (*p* < 0.05) and remained significantly elevated for 72 h after the match (*p* < 0.05). Muscle damage biomarkers peaked at 24 h (*p* < 0.05) and remained significantly (*p* < 0.05) elevated for at least 72 h after the match. Oxidative stress markers peaked at 24 h–48 h (*p* < 0.05) and returned to baseline 120 h post-match (*p* > 0.05). In respect to antioxidant responses, these peaked at 24 h–48 h post-match (*p* < 0.05) and returned to baseline 120 h after the match (*p* > 0.05). Conclusions: A single futsal match induces short/mid-term changes in performance, inflammation, oxidative stress, and muscle damage markers for about 72 h–96 h post-match.

## 1. Introduction

Futsal, as a five-a-side indoor soccer, is an intermittent, high-intensity, relatively new sport, that is steadily growing in popularity [1]. It requires special tactics, and is characterized by high technical skills and elevated physical demands [2]. During a futsal match, players typically cover 4–5 km, with more than 50% of that distance at intensities >80–85% of their maximal heart rate (HR_max_) [3], blood lactate concentration exceeds 5 mmol·L^−1^ [4], and during professional matches the intensity in correspondence to oxygen uptake ranges from 50 to 55 mL·kg^−1^·min^−1^ [5]. As it is understood, such a demanding sport requires a combination of high aerobic and anaerobic capacities to achieve an optimal performance [3,4,6]. However, limited research has been conducted in this sport [1,5] and especially in female athletes [1].

Futsal players execute a great number of repeated high-intensity actions (e.g., rapid changes in direction, accelerations and decelerations, jumps, physical contact with other athletes), which may expose them to high neuromuscular fatigue [2]. Fatigue affects muscle function and results in reduced performance, possible for several days after a futsal match in male [7] and female [8,9] players. It has been found that repetitive eccentric muscle activities are related to soccer-match-induced muscle damage both in males and females [10,11], delayed onset muscle soreness (DOMS), and deterioration of performance due to the applied strain forces on skeletal muscle and deep fascia [12]. However, the underlying mechanisms associated with transient inflammatory responses are multidimensional and are influenced by central and peripheral regulatory pathways [13]. Several studies have also shown that high-intensity exercise may induce acute or chronic muscle injuries, and the release of cytosolic enzymes in the bloodstream, such as lactate dehydrogenase (LDH) and creatine kinase (CK), mainly its skeletal muscle isoform CK-MM, which may be considered as indexes of cellular lesions [14,15,16]. Creatine kinase and LDH biomarkers are related with muscle inflammation and micro-injuries, causing a delayed increment on inflammatory markers in the bloodstream, such as C-reactive protein (CRP), after a high-level competitive soccer match [17]. Moreover, inflammatory response to cellular lesions is initiated by leukocytes, plasma protein, and fluid infiltration into the muscle, while cytokines and chemokines pertain in the onset of these responses [15,18,19]. In addition to this, the elevated production of inflammatory mediators (such as cytokines), reactive oxygen species, and phagocyte infiltration into the muscle, accelerate the muscle damage process [20]. Although, many of the acute substrate oxidation changes that take place during a soccer match return to their normal range during the post-match recovery, considerable changes have been observed also for up to 2–3 days post-match [21]. This is why soccer practitioners employ recovery strategies when implementing training sessions, to speed up recovery and effectively prepare players for the following match [22].

Futsal, on one hand, is characterized by high-intensity efforts of ~2–3 s [6] with brief recovery periods of ~20–30 s, in association with constant changes in speed and direction [6,23], making the intensity of futsal appear to be more pronounced than that of a soccer match [21]. In this sense, taking into account the metabolic demands of the futsal match, the internal and external training load requirements, and more importantly the need for adequate recovery after strenuous exercise so as not to jeopardize the quality of the training schedule, the weekly microcycles may also needed to include sufficient recovery periods and/or tapering practices between competitive matches [24]. On the other hand, despite the fact that the number of futsal-related investigations has increased in recent years [1] and although physiological, hematological, biochemical, and inflammatory response changes associated with competitive matches and/or exercise have been extensively examined in soccer [11,25,26,27,28], to our knowledge studies that evaluate oxidative stress, inflammation, and muscle damage markers as the consequences of a match during a whole weekly microcycle in futsal (to support coaches’ interventions) are very scarce [1,13,21] and even less in female futsal athletes [29,30]. Moreover, since males and females have biological differences that result in different physiologic responses to exercise [31], it highlights the athletic trainers’ need to comprehend whether a women’s futsal match can potentially induce muscle damage and induce changes in oxidative stress products as well. That stresses even more the need to examine the time course of performance indexes, oxidative stress, inflammation, and muscle damage markers in female athletes after a futsal match, so that (i) sport trainers are able to perceive in time the fatigue that players may experience [32], and (ii) to assist sports trainers in taking immediate action (i.e., applied strategies for recovery and improvement of physical performance after a female match) to prevent potential overreaching and excessive training during the weekly training microcycle. Therefore, the aim of the current study was to evaluate the potential time-course changes in several oxidative stress, inflammation, muscle damage markers, and performance indexes before and immediately after an unofficial futsal match, and during the next six consecutive days in female athletes regardless of the position they play (defenders, wingers, pivots).

## 2. Materials and Methods

### 2.1. Participants

**Figure 1:**
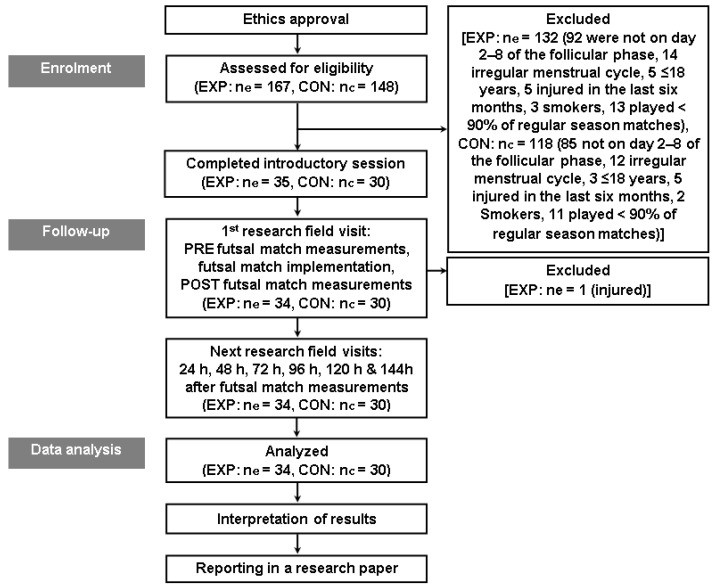
Experimental design.

Written consent was obtained from the potential participants after they were thoroughly informed of the aims, procedures, benefits, and potential risks of participating in the study. Prior to the study (Figure 1), all potential participants filled out medical, smoking and sleeping habits, and physical activity questionnaires [33,34,35,36] and self-reported their menstrual cycle phases. The participant selection was based on the following inclusion criteria: female non-smokers national-level futsal players (tier 3) [37] and highly physically active (>1000 MET-min·week ^−1^) [38] that had played at least 90% of the regular season matches and at least 20 min per match as defender, midfielder, or attacker, >18 years old, free from injury or illness, not on medication and alcoholic products during the last six months, regular menstrual cycle, no history of menstrual problems, no use of hormonal contraception for at least 6 months prior to testing, and no participation in other sport activities during the study. Moreover, during the intervention of an unofficial futsal match, all participants had to be in the follicular phase of their menstrual cycle (i.e., 2–8th day after the onset of menstruation) when estrogen and progesterone concentrations are low [39] and perceived or objective athletic performance does not seem to be negatively impaired in eumenorrheic female athletes [40]. All procedures and experimental tests were conducted according to the Declaration of Helsinki guidelines [41] and were approved by the Ethics Committee of the School of Physical Education and Sport Science, National and Kapodistrian University of Athens, Greece (1266/17-2-2021).

The participants were recruited from women’s futsal top national leagues, first (12 clubs) and second division (12 clubs), and all registered players who competed in these two categories were informed about the study. The 167 players from the first division who expressed their desire to participate were considered as potential participants of the experimental group. However, 92 (55.09%) were not on day 2–8 of the follicular phase of their menstrual cycle during the upcoming intervention of futsal match and served only as co-players or goalkeepers or substitutes in case of need and were excluded from the experimental group. From the remaining players, 40 (23.95%) also did not meet the criteria for inclusion (14 irregular menstrual cycle, 5 ≤ 18 years, 5 injured in the last six months, 3 smokers, 13 played < 90% of regular season matches) and were thus excluded. Next, using a computer-generated list of random numbers, the remaining 35 potential participants were randomly assigned as defenders, midfielders, or attackers in 10 different teams where the goalkeepers and the remaining positions were randomly held by healthy previously excluded players. Consequently, five matches were held in parallel in a multi-facility sports center. Potential participants had to play the entire duration of a match (two periods of 20 min, pitch dimension: 38 m × 20 m, surface of the pitch: wood), while at the researcher’s signal they would regularly change their field position in a circular manner to ensure that each participant played approximately the same amount of time in all positions (defenders, wingers, pivots). Thus, excluding the players who were used to complete the residual positions of the teams and one potential participant who was injured during the intervention of the futsal match, who was also excluded, the remaining players (20.36%) form the experimental group of the study (EXP: ne = 34, age = 22.6 ± 2.1 years, height = 167.4 ± 3.6 cm, body mass = 57.5 ± 3 kg). The 148 players from the second division who expressed their desire to participate were considered as potential participants of the control group. However, 118 (79.73%) did not meet the criteria for inclusion (85: not on day 2–8 of the follicular phase, 12: irregular menstrual cycle, 3: ≤18 years, 5: injured in the last six months, 2: smokers, 11: played < 90% of regular season matches) and were excluded. The remaining players (20.27%) were selected for the control group (CON: nc = 30, age = 22.3 ± 2.6 years, height = 166.6 ± 4.6 cm, body mass = 56.8 ± 3.4 kg).

### 2.2. Procedures

Based on the biomarkers empirical evidence derived from the EROS study [42] and their importance to the authors, several oxidative stress, inflammation, subjective muscle soreness, subjective rate perceived exertion, and performance tests were conducted before (PRE) and immediately after a futsal match (POST), as well as 24 h, 48 h, 72 h, 96 h, 120 h, and 144 h post-match. The same measurements at the same time were collected from the control group without them participating in any form of physical activity. Regardless of the group allocation, all participants were instructed to perform at the best of their ability and no feedback was given until the end of the study. The control group did not know that the experimental group had played a futsal match and vice versa. Both the participants and the assessment researchers were unaware of the real purposes of the research (double blinded design). The futsal match was played at an indoor court in accordance with the international futsal regulations and eleven days after the last day of the regular season (September–May). In this week, prior to the match intervention, the players did not consume any ergogenic or synergistic substances [43,44,45], abstained from any heavy training load, and participated only in recovery training sessions in order to practice on regular game tactics and team cohesion. The match was performed in June and during the afternoon (17:00 PM), with mean outdoor and indoor temperatures, humidity, and barometric pressure range of 25 °C, 23 °C, 35%, and 1010–1025 mmHg, respectively. During the week before the match, the participants were instructed to follow a balanced diet, i.e., 50–60% carbohydrates energy intake, 25–30% fat, and 15% protein, and to record their diet content (i.e., ingredients and portions) in as much detail as possible. The participants were also asked to replicate their prerecorded diet during the 6 days after the match as accurately as possible. On the match day, all players consumed a certain meal (plain pasta: 100 g, grilled chicken breast: 180 g, and a typical medium-sized banana: ~100 g) rich in carbohydrates (approximately 65% of total energy intake) 4.5 h before the start of the match. Water was provided ad libitum before, during half-time, and at the end of the match. Following baseline blood sampling that was taken between 08.00 and 10.00 A.M., the athletes consumed a light, standardized pre-match meal that was utilized to control for micronutrients, vitamins, and selenium intake. Moreover, in the week after the match all participants abstained from any structured physical activities and were advised to engage in light activities for recovery purposes.

### 2.3. Measurements

#### 2.3.1. Menstrual Cycle

The participants’ phases of the menstrual cycle were self-identified after the detection of the luteinizing hormone (LH) surge using the Clearblue Digital Ovulation Test^®^ (Clearblue, Geneva, Switzerland). The participants were instructed to begin using the test daily starting from the first day of their menstrual cycle. The test detects the LH surge, which typically occurs 24–36 h before ovulation. The participants were advised to interpret the test results according to the manufacturer’s instructions and classify their menstrual cycle phases based on the observed LH surge. They were provided with a standardized guide that outlined the criteria for identifying each phase, including the follicular, ovulatory, and luteal phases. The self-identification of the menstrual cycle phases was conducted in a private and confidential manner to ensure accurate reporting. The test strips and instructions were provided to participants at the beginning of the study, and they were asked to record the date of the LH surge and the corresponding cycle phase in a provided diary. This approach allowed for the collection of menstrual cycle data while minimizing interference with the participants’ daily routines. Additionally, the participants were informed about the potential limitations of self-identification and were encouraged to consult with healthcare professionals for confirmation or further guidance if needed.

#### 2.3.2. Anthropometric Assessment

An anthropometric assessment was conducted during a preliminary visit to the laboratory one week pre-match. Standing height to the nearest 0.5 cm (Stadiometer^®^, Seca, Birmingham, UK) and nude body mass to the nearest 0.1 kg (Beam balance 710; Seca, Birmingham, UK) were measured. Harpenden Skinfold Calipers^®^ (Baty International, West Sussex, UK) were used to measure the thickness of seven skinfolds [24] and body fat (BF) was estimated using relevant equations [46].

#### 2.3.3. Maximal Oxygen Uptake and Heart Rate

Maximal oxygen uptake (⩒O_2max_) was measured in a laboratory one week pre-match and after a preliminary familiarization visit for ⩒O_2max_ assessments and lower limb muscle strength tests, as well as sprint performance tests. For the measurement of ⩒O_2max_, a portable gas exchange analyzer (K4b^2®^, Cosmed, Rome, Italy) that had been calibrated with known oxygen and carbon dioxide gas mixtures and an incremental running test to exhaustion on a treadmill (Technogym Runrace^®^, Technogym, Gambettola, Italy) were used. The ⩒O_2max_ protocol consisted of running at 7 km·h^−1^ for 1 min and 8 km·h^−1^ for 30 s. Thereafter, the speed of the treadmill was increased by 0.5 km·h^−1^ every 30 s until exhaustion; the inclination of the treadmill was 1%. The ⩒O_2max_ and HR_max_ were determined as the highest values reached in a 15 and 5 s period, respectively, during the last part of the incremental test. The criteria for the attainment of ⩒O_2max_ included two of the following: a respiratory exchange ratio >1.1, HR_max_ within 10 bpm of the estimated HR_max_ based on age, a rating of perceived exertion equal to, or higher than 18, or a leveling off in ⩒O_2_ (<2 mL·kg^−1^·min^−1^) with a concomitant increase in treadmill speed [47]. Heart rate (HR) was measured telemetrically (Polar FT1 Model^®^, Polar Vantage NV monitor; Polar Electro Oy, Kempele, Finland). Throughout the futsal match HR was recorded at 5 s intervals.

#### 2.3.4. Lower Limb Power and Sprint Performance

The participants’ lower limb power was measured with counter movement jump (CMJ) with arm swing on a EuroJump^®^ contact platform (Newtest, Oulu, Finland) after a standardized 5 min warm up at 60–70% intensity on a Monark leg cycle ergometer (894E^®^, Monark, Varberg, Sweden) [48]. The sprint test consisted of a 20 m straight line track with 10 m split time recording, which was measured by three pairs of infrared light sensors (Powertimer 300-series^®^, Newtest, Oulu, Finland) placed at a 100 cm height. The tests were conducted from a standing position, with the front foot placed 30 cm behind the start line.

#### 2.3.5. Subjective Muscle Soreness and Fatigue Measurements

For leg dominance determination, the Waterloo Footedness Questionnaire-Revised was used [49]. Next, each participant, after three squat positions and through 3 s of palpation, was asked to complete a muscle soreness questionnaire, in which they rated their perceived DOMS on a scale from 0 (normal absence of soreness) to 10 (very intense sore) for knee extensor (KE) and flexor (KF) muscles (quadriceps, hamstrings, gastrocnemius, and tibialis) for both dominant (D) and non-dominant (ND) legs [50]. The self-recorded subjective muscle soreness was used as an indicator of the muscle soreness of the players. Furthermore, the subjective rate perceived exertion (RPE) was estimated using the 6–20 Borg scale [51] and was used as an indicator of muscle exertion and fatigue.

#### 2.3.6. Blood Sampling and Assays

Blood drop samples were collected within 1 min before the match, immediately after the 1st half, before the 2nd half, during half-time, and within 1 min after the completion of the match to measure lactate concentration (Accutrend Plus^®^, F. Hoffmann-La Roche Ltd., Rotkreuz, Switzerland). Moreover, eight blood samples in total per participant were drawn from the basilic or mesobasilic vein with the player in a sitting position in the morning after a 12 h fasting before the match day, immediately after the match, and at 24, 48, 72, 96, 120, and 144 h post-match. Approximately 10 mL from each sample was treated with sodium citrate, centrifuged for 10 min at 2250× *g*, and the supernatant plasma was stored at −70 °C for later analyses. Approximately 5 mL of the sample was placed in vacutainers without anticoagulant, left to clot, and then centrifuged at 2250× *g* for 15 min at 4 °C. The serum samples obtained were separated from the packed red cells, placed in Eppendorf tubes, and stored at −70 °C. A small quantity of blood (0.2 mL), which was immediately added to trichloroacetic acid (0.4 mL of 5%) and centrifuged (2500× *g*, 15 min), was analyzed spectrophotometrically at the 412 nm wavelength for reduced glutathione (GSH) and oxidized glutathione (GSSG) determination, as described in previous studies [52,53]. Using the JEOL ion exchange chromatography method, homocysteine (HCY) was measured by photometric detection (JEOL AminoTac JLC500/V analyser^®^, Akishima, Tokyo, Japan) after a reaction with ninhydrin, as described in detail by Suen et al. [54]. The determination of cortisol, interleukin-6 (IL-6), and tumor necrosis factor alpha (TNF-α) was performed using commercially available ELISA kits (E-EL-0157/E-EL-H6156/E-EL-H0109^®^, Elabscience, Houston, TX, USA) according to the manufacturer’s instructions on a standard ELISA reader (Spark 10M^®^, Tecan, Mannedorf, Switzerland). The levels of CRP were determined immunonephelometrically on a biochemistry analyzer (COBAS e411^®^, F. Hoffmann-La Roche Ltd., Rotkreuz, Switzerland) according to the manufacturer’s directions. On an MDA180^®^ automated analyzer (bioMerieux, Lyon, France), fibrinogen (FIB) was measured using a functional assay based on Clauss’ method and the kit Fibriquik (bioMerieux, Lyon, France), as described elsewhere [55,56]. Commercially available kits (CK test strips^®^, Roche, Basel, Switzerland) and a reflectance photometer (Reflotron Plus System^®^, Roche, Basel, Switzerland) were used to measure CK. Thiobarbituric acid reactive substances (TBARSs), protein carbonyls (PCs), activity of serum catalase (CAT), and total antioxidant capacity (TAC) were analyzed spectrophotometrically at 530 nm, 395 nm, 240 nm, 540 nm wavelengths (Hitachi 917^®^ analyzer, F. Hoffmann-La Roche Ltd., Rotkreuz, Switzerland) according to Keles et al., Patsoukis et al., Aebi, and Janaszewska and Bartosz, respectively [57,58,59,60]. The activity of LDH was measured spectrophotometrically using a commercial diagnostic test kit (MAK066^®^, Sigma Diagnostics, St. Louis, MO, USA). Uric acid (UA) and complete white blood cell count (WBC) was determined within 24 h via matching duplicate counts using an automated hematology analyzer (Sysmex K-1000^®^, TOA Electronics, Hyogo, Japan). All samples were measured in duplicate and their average was used for statistical analysis (in all assays performed, the interassay and intraassay coefficients of variation ranged from 2.4 to 8.7 and 3.5 to 8.9, respectively, according to the manufacturers’ internal studies).

### 2.4. Statistical Analyses

The homogeneity and normality of the obtained data were assessed through the Levene’s and Shapiro–Wilk tests, respectively. Independent *t*-tests were used to compare the pre-match descriptive variables between groups. Changes across different time measurements between groups were assessed with a 2 by 8 (groups × time) mixed analysis of variance (ANOVA) with repeated measures on the time factor. A 2 by 3 and a 2 by 5 (groups × time) mixed ANOVA with repeated measures on the time factor was conducted for the heart rate and lactate measurements. The degrees of freedom (df) for the main effects, interactions, and error terms were adjusted according to Greenhouse–Geisser correction when the assumption of sphericity was violated. For statistically significant interactions, post hoc analyses of simple effects and analytical pairwise comparisons with Bonferroni correction were used to identify significant differences [61] between time measurements for the experimental group. Effect sizes were estimated by calculating partial eta squared (η_p_^2^). The participants’ pooled values (i.e., values per match) of blood lactate concentration after the match for the five matches were analyzed with a one-way ANOVA design (1st match × 2nd match × 3rd match × 4th match × 5th match). The Pearson’s correlation coefficient, r, was used to evaluate the intercorrelations among the biochemical and performance variables. All data were analyzed with Statistical Package for the Social Sciences for Windows (SPSS 26.0, IBM Corp, Armonk, NY, USA) and according to the procedures outlined by Meyers et al. [62]. Statistical significance was set at *p* < 0.05 for all analyses. Partial η^2^ and Pearson product moment correlations (r) were calculated and interpreted according to Cohen (1988) [63]. GraphPad StatMate Version 2.0 (GraphPad Software Inc., La Jolla, CA, USA) was used to calculate post hoc power using TBARSs as a criterion variable. The following parameters were used: an effect size of 0.93 (η_p_^2^), a sample size of 64, two groups in two conditions, and eight repeated measures. The observed power (1 − β) was >0.9. When other power variables (e.g., CK, CRP, WBC, and LDH) were used, a similar power was calculated.

## 3. Results

The descriptive characteristics (see Table 1) and pre-match measurements did not show statistical significance between the groups (*p* > 0.05). Additionally, time measurements did not show statistical significance for the control group in all variables (*p* > 0.05). Therefore, for significant interactions, statistical findings are presented based on simple effects (F-values with corrected df) and Bonferroni pairwise comparisons between time measurements for the experimental group in all dependent variables. Moreover, as reflected by the after-match blood lactate concentration measurements, participants’ match intensity of the five played matches showed no statistically significant difference between matches as determined by one-way ANOVA (F(4, 29) = 0.084, *p* = 0.987). 

### 3.1. Performance Assessments (Figure 2)

#### 3.1.1. Counter Movement Jump

The results of simple effects analysis on the CMJ measurements for the experimental group showed statistically significant differences (F(2.04, 67.44) = 137.28, *p* < 0.001, η_p_^2^ = 0.81). Bonferroni pairwise comparisons indicated that the lowest CMJ value occurred immediately after the game (*p* < 0.05), it remained significantly lower than the PRE measurement, 24 h and 48 h after the match (*p* < 0.05), and returned to baseline levels 72 h after the match. Interclass correlations showed that CMJ 48 h after the match was negatively correlated with TNF-α, cortisol, and WBC (r = −0.41, *p* < 0.05; r = −0.34, *p* < 0.05; r = −0.47, *p* < 0.01, respectively), whereas it was positively correlated with HCY (r = 0.40, *p* < 0.05). Interclass correlations of the significant differences between the deficit of pre- and post-match performance measurements showed that CMJ was positively correlated with pre-match HCY (r = 0.37, *p* < 0.05), negatively with pre-match CK (r = −0.39, *p* < 0.05), and positively with the 24 h GSH/GSSG ratio (r = 0.39, *p* < 0.05).

**Figure 2:**
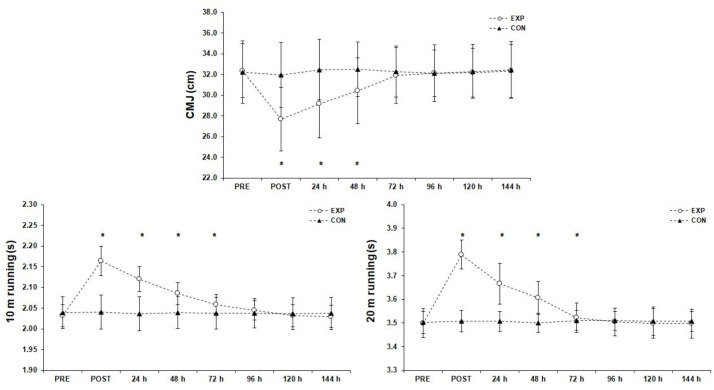
Performance assessments before (PRE), after (POST), and for six consecutive days after the futsal match (mean ± SD). * Significant difference from PRE measurements in experimental group (*p* < 0.05). Abbreviations: CMJ, counter movement jump; CON, control group; EXP, experimental group; SD, standard deviation.

#### 3.1.2. 10 m and 20 m Sprint

The results of the simple effects analyses indicated significant differences for the 10 m sprint (F(1.96, 64.81) = 1008.95, *p* < 0.001, η_p_^2^ = 0.97) and for the 20 m sprint (F(1.63, 53.81) = 2069.01, *p* < 0.001, η_p_^2^ = 0.98) measurements. Bonferroni pairwise comparisons showed that the 10 m sprint had the highest time scores immediately after the match (*p* < 0.05) and these remained significantly higher than the PRE measurement, 24 h, 48 h, and 72 h after the match (*p* < 0.05). Similarly, the 20 m sprint had the highest time scores immediately after the match (*p* < 0.05) and it remained significantly higher than the PRE measurement, 24 h, 48 h, and 72 h after the match (*p* < 0.05). Furthermore, the 10 m and 20 m sprint times returned almost to baseline 120 h and 96 h after the match, respectively. Interclass correlations of the significant differences between the deficit of pre- and post-match performance measurements showed that the 10 m sprint was negatively correlated with pre-match, 24 h, and 96 h after the match IL-6 (r = −0.34, *p* < 0.05; r = −0.36, *p* < 0.05; r = −0.38, *p* < 0.05, respectively), positively with pre-match and post-match LDH (r = 0.50, *p* < 0.01 and r = 0.52, *p* < 0.01, respectively), positively with 24 h after the match HCY (r = 0.38, *p* < 0.05), and positively with post-match and 48 h, 72 h, and 96 h after the match FIB (r = 0.36, *p* < 0.05; r = 0.45, *p* < 0.01; r = 0.44, *p* < 0.01; r = 0.41, *p* < 0.05, respectively). Similarly, the deficit of the pre- and post-match 20 m sprint was negatively correlated with post-match IL-6 (r = −0.42, *p* < 0.05).

### 3.2. Subjective Muscle Soreness and Fatigue Measurements (Figure 3)

#### 3.2.1. Delayed Onset Muscle Soreness

Statistical analyses indicated significant differences for DOMS among measurements of the experimental group for the dominant leg extensors (DKE) (F(1.68, 55.56) = 792.10, *p* < 0.001, η_p_^2^ = 0.96), non-dominant leg extensors (NDKE) (F(2.21, 72.77) = 802.06, *p* < 0.001, η_p_^2^ = 0.96), dominant leg flexors (DKF) (F(2.38, 78.39) = 939.53, *p* < 0.001, η_p_^2^ = 0.97), and non-dominant leg flexors (NDKF) (F(2.92, 96.25) = 887.33, *p* < 0.001, η_p_^2^ = 0.96). Bonferroni pairwise comparisons showed that (i) both DKE and NDKE DOMS values peaked 24 h after the match, were significantly higher than the PRE measurements post-match, and 48 h and 72 h after the match (*p* < 0.05), and returned to baseline 96 h after the match, and (ii) both DKF and NDKF DOMS peaked 24 h after the match, were significantly higher than the PRE measurement post-match, and 48 h, 72 h, and 96 h after the match (*p* < 0.05), and returned to baseline 120 h after the match. Interclass correlations of the significant differences between the deficit of the pre- and post-match DOMS measurements showed that DKE was positively correlated with pre-match and post-match HCY (r = 0.35, *p* < 0.05 and r = 0.36, *p* < 0.05, respectively), as well as with pre-match WBC (r = 0.35, *p* < 0.05). On the other hand, NDKE was negatively correlated with pre-match HCY (r = 0.36, *p* < 0.05), whereas it was positively correlated with post-match LDH (r = −0.45, *p* < 0.01). Regarding the DKF pre- and post-match deficit, a negative correlation was found with post-match UA and TBARSs 72 h after the match (r = −0.35, *p* < 0.05 and r = −0.35, *p* < 0.05, respectively). Finally, the NDKF pre- and post-match deficit was negatively correlated with the pre-match and post-match UA (r = −0.36, *p* < 0.05 and r = −0.43, *p* < 0.05, respectively), as well as with CK 24 h and 48 h after the match (r = −0.36, *p* < 0.05 and r = −0.40, *p* < 0.05, respectively) and positively with WBC 72 h after the match (r = 0.43, *p* < 0.05).

**Figure 3:**
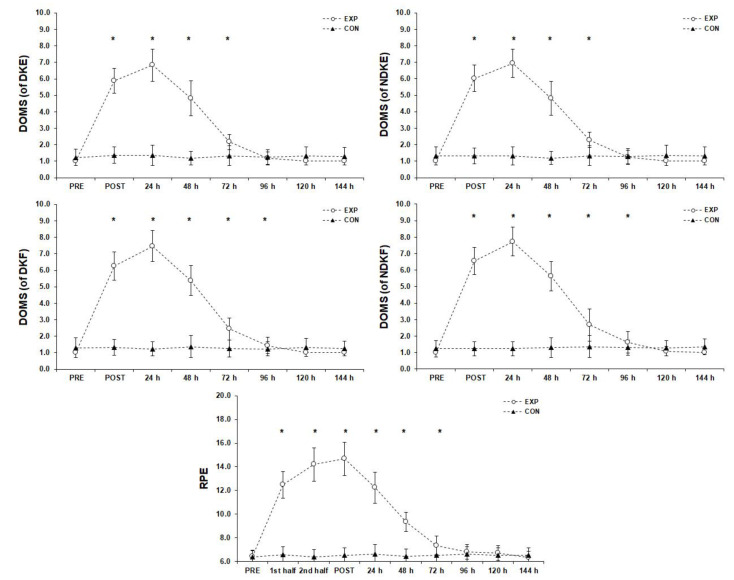
Subjective muscle soreness and fatigue measurements before (PRE), during (1st and 2nd half), after (POST), and six days after the match (mean ± SD). * Significant difference from PRE measurements in experimental group (*p* < 0.05). Abbreviations: CON, control group; EXP, experimental group; DOMS, delayed onset muscle soreness; DKF, dominant leg flexors; DKE, dominant leg extensors; NDKE, non-dominant leg extensors; NDKF, non-dominant leg flexors; RPE, rate of perceived exertion; SD, standard deviation.

#### 3.2.2. Rate of Perceived Exertion

Statistically significant changes (F(3.34, 110.07) = 688.58, *p* < 0.001, η_p_^2^ = 0.95) were found among the RPE measurements of the experimental group. Bonferroni pairwise comparisons showed that RPE peaks occurred immediately after the match (*p* < 0.05) and were significantly higher after the 1st half, before the 2nd half, and 24 h, 48 h, and 72 h after the match (*p* < 0.05). The RPEs returned to baseline 120 h after the match. Interclass correlations of the significant differences between the deficit of the pre- and post-match measurements showed that the RPE was positively correlated with CK pre-match and 72 h after the match (r = 0.36, *p* < 0.05 and r = 0.37, *p* < 0.05, respectively). On the other hand, RPE was negatively correlated with FIB post-match and 24 h after the match (r = −0.38, *p* < 0.05 and r = −0.61, *p* < 0.01, respectively), and with CRP 48 h and 72 h after the match (r = −0.36, *p* < 0.05 and r = −0.39, *p* < 0.05, respectively).

### 3.3. Heart Rate and Blood Lactate Measurements (Figure 4)

#### 3.3.1. Heart Rate

There was a significant difference (F(1.77, 58.40) = 143,172.82, *p* < 0.001, η_p_^2^ = 1.00) for the HR measurements of the experimental group and Bonferroni pairwise comparisons indicated that the HR values were similarly high during the 1st and the 2nd half and significantly different to pre-match values (*p* < 0.05). 

**Figure 4:**
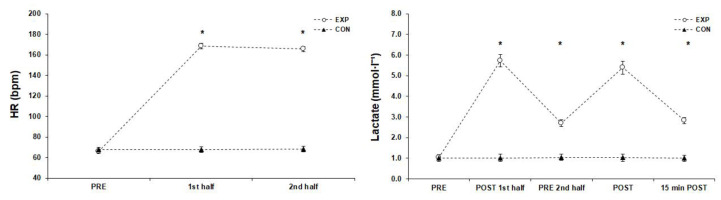
Match intensities before (PRE), during (1st and 2nd half), after (POST), and 15 min after the match (mean ± SD). * Significant difference from PRE measurements in experimental group (*p* < 0.05). Abbreviations: CON, control group; EXP, experimental group; HR, heart rate; SD, standard deviation.

#### 3.3.2. Lactate

The simple effects analysis for blood lactate concentration indicated significant difference among the measurements (F(1.80, 59.35) = 3749.49, *p* < 0.001, η_p_^2^ = 0.99) and Bonferroni pairwise comparisons showed that the lactate peak occurred immediately after the 1st and the 2nd half (*p* < 0.05), and remained significantly higher than the pre-match values before the 2nd half as well as 15 min after the match. 

### 3.4. Inflammatory Responses (Figure 5)

#### 3.4.1. Interleukin-6

The simple effects analysis showed a significant difference among IL-6 responses (F(1.12, 36.83) = 1538.31, *p* < 0.001, η_p_^2^ = 0.98) and Bonferroni post hoc analysis showed that IL-6 peaked immediately after the match and returned to baseline in the next morning (*p* < 0.05). 

**Figure 5:**
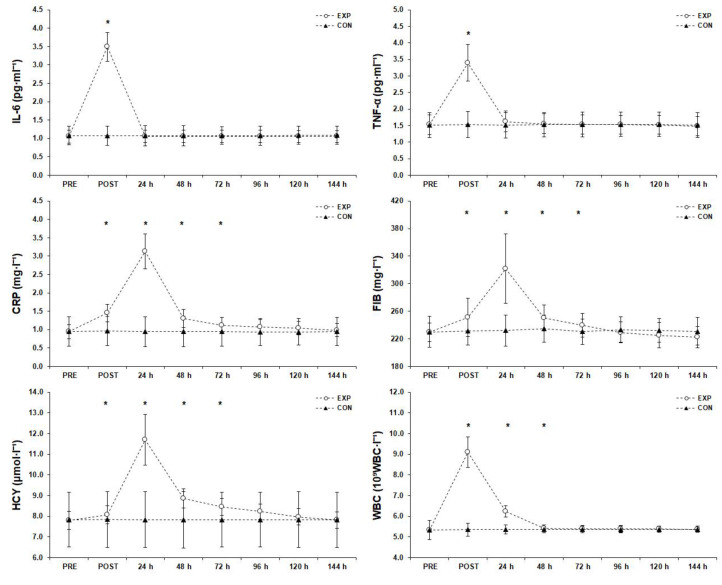
Inflammatory responses before (PRE), after (POST), and 6 days after the match (mean ± SD). * Significant difference from PRE measurements in experimental group (*p* < 0.05). Abbreviations: CON, control; CRP, C-reactive protein; EXP, experimental group; FIB, fibrinogen; HCY, homocysteine; IL-6, interleukin-6; SD, standard deviation; TNF-α, tumor necrosis factor alpha; WBC, white blood cell count.

#### 3.4.2. Tumor Necrosis Factor alpha

A significant difference (F(1.98, 65.31) = 217.43, *p* < 0.001, η_p_^2^ = 0.87) was found for the TNF-α measurements and post hoc analysis showed that TNF-α peaked immediately after the match (*p* < 0.05) and returned almost to baseline by the next morning. 

#### 3.4.3. C-Reactive Protein

Statistical significance (F(1.89, 62.20) = 358.23, *p* < 0.001, η_p_^2^ = 0.92) was found for CRP and post hoc analysis indicated that the CRP peak occurred 24 h after the match (*p* < 0.05), remained significantly higher than the PRE measurement post-match and 48 h and 72 h after the match, and returned almost to baseline 96 h after the match. 

#### 3.4.4. Fibrinogen

Statistical analysis showed a significant difference (F(1.81, 59.57) = 85.42, *p* < 0.001, η_p_^2^ = 0.72) for the FIB measurements. Post hoc analysis showed that the FIB peak occurred 24 h after the match, remained significantly higher than the PRE measurement post-match and 48 h and 72 h after the match (*p* < 0.05), and returned almost to baseline 96 h after the match. 

#### 3.4.5. Homocysteine

There was a significant difference (F(1.60, 52.70) = 280.15, *p* < 0.001, η_p_^2^ = 0.89) for HCY and post hoc analysis showed that the HCY peak occurred 24 h after the match, was significantly higher than the PRE measurement post-match and 48 h and 72 h after the match (*p* < 0.05), and reached baseline values 96 h after the match.

#### 3.4.6. White Blood Cell Count

A significant difference (F(1.17, 38.54) = 775.96, *p* < 0.001, η_p_^2^ = 0.96) was found for the WBC measurements. A post hoc analysis showed that WBC peaked immediately after the match and was higher than the PRE measurement 24 h and 48 h after the match (*p* < 0.05), and returned almost to baseline 72 h after the match (*p* < 0.05).

### 3.5. Muscle Damage Markers (Figure 6)

#### 3.5.1. Lactate Dehydrogenase

The simple effects analysis showed a significant difference among measurements (F(3.74, 123.32) = 909.19, *p* < 0.001, η_p_^2^ = 0.88) of LDH. Pairwise comparisons revealed that the LDH peak occurred 24 h after the match, was significantly higher than the PRE measurement post-match and 48 h and 72 h after the match (*p* < 0.05), and returned almost to baseline 96 h after the match.

**Figure 6:**
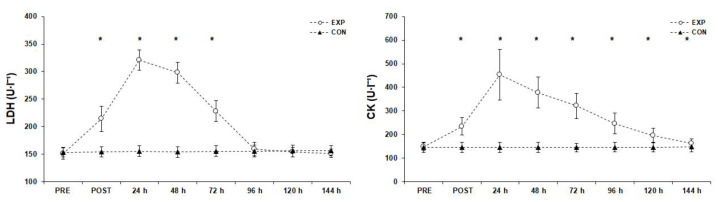
Muscle damage measurements before (PRE), after (POST), and 6 days after the match (mean ± SD). * Significant difference from PRE measurements in experimental group (*p* < 0.05). Abbreviations: CK, creatine kinase; CON, control group; EXP, experimental group; LDH, lactate dehydrogenase; SD, standard deviation.

#### 3.5.2. Creatine Kinase

Statistical analysis showed a significant difference (F(1.89, 62.46) = 244.69, *p* < 0.001, η_p_^2^ = 0.88) for the CK measurements. A post hoc analysis showed that CK peaked 24 h after the match and remained significantly higher than the PRE measurement post-match and 48 h, 72 h, 96 h, 120 h, and 144 h after the match (*p* < 0.05).

### 3.6. Oxidative Markers (Figure 7)

#### 3.6.1. Protein Carbonyls

The simple effects analysis for PC concentration revealed significant difference among the measurements (F(2.48, 81.74) = 72.94, *p* < 0.001, η_p_^2^ = 0.69) and post hoc analysis showed that PCs peaked 48 h after the match, remained significantly higher than the PRE measurement post-match, and 24 h, 72 h, and 96 h after the match (*p* < 0.05), and returned to baseline levels 120 h after the match.

**Figure 7:**
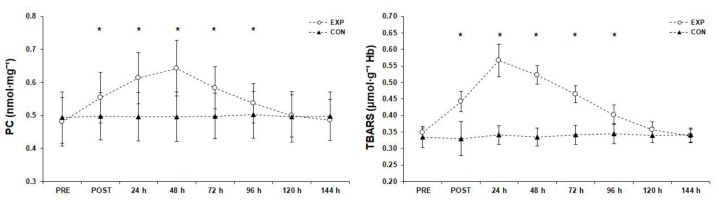
Oxidative markers before (PRE), after (POST), and 6 days after the match (mean ± SD). * Significant difference from PRE measurements in experimental group (*p* < 0.05). Abbreviations: CON, control group; EXP, experimental group; PCs, protein carbonyls; SD, standard deviation; TBARSs, thiobarbituric acid reactive substances.

#### 3.6.2. Thiobarbituric Acid Reactive Substances

A significant difference (F(2.62, 86.56) = 431.05, *p* < 0.001, η_p_^2^ = 0.93) was found for the TBARSs measurements and post hoc analysis showed that TBARSs peaked 24 h after the match, remained significantly higher than the PRE measurement post-match and 48 h, 72 h, and 96 h after the match (*p* < 0.05), and returned to baseline 120 h after the match.

### 3.7. Antioxidant Responses (Figure 8)

#### 3.7.1. Reduced Glutathione

The simple effects analysis for GSH concentration showed a significant difference among the measurements (F(1.86, 61.29) = 125.56, *p* < 0.001, η_p_^2^ = 0.79). A post hoc analysis showed that the lowest GSH value occurred 24 h after the match, was significantly lower than the PRE measurement post-match and 48 h after the match (*p* < 0.05), and returned to baseline 72 h after the match.

**Figure 8:**
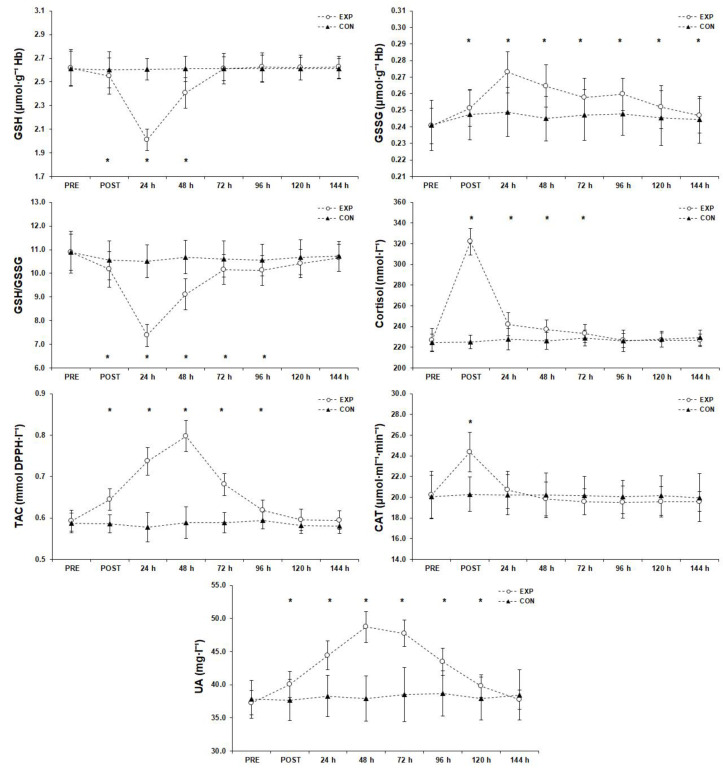
Antioxidant responses before (PRE), after (POST), and 6 days after the match (mean ± SD). * Significant difference from PRE measurements in experimental group (*p* < 0.05). Abbreviations: CAT, catalase; CON, control group; EXP, experimental group; GSH, reduced glutathione; GSSG, oxidized glutathione; GSH/GSSG, reduced glutathione/oxidized glutathione; TAC, plasma total antioxidant capacity; SD, standard deviation; UA, uric acid.

#### 3.7.2. Oxidized Glutathione

A significant difference (F(3.88, 128.01) = 82.85, *p* < 0.001, η_p_^2^ = 0.72) was found for the GSSG measurements and post hoc analysis showed that GSSG peaked 24 h after the match and remained significantly higher than the PRE measurement post-match and 48 h, 72 h, 96 h, 120 h, and 144 h after the match (*p* < 0.05).

#### 3.7.3. GSH/GSSG Ratio

Statistical significance (F(2.47, 81.54) = 165.21, *p* < 0.001, η_p_^2^ = 0.83) was found for the GSH/GSSG ratio and post hoc analysis indicated that the lowest GSH/GSSG ratio value occurred 24 h after the match, was significantly lower than the PRE measurement post-match and 48 h, 72 h, and 96 h after the match (*p* < 0.05), and returned to baseline 120 h after the match.

#### 3.7.4. Cortisol

Statistical analysis showed a significant difference (F(2.70, 89.19) = 747.03, *p* < 0.001, η_p_^2^ = 0.96) for the cortisol measurements. A post hoc analysis revealed that cortisol peaked immediately after the match, was significantly higher than the PRE measurement 24 h, 48 h, and 72 h after the match (*p* < 0.05), and returned to baseline 96 h after the match.

#### 3.7.5. Plasma Total Antioxidant Capacity

There was a significant difference (F(1.60, 52.70) = 762.60, *p* < 0.001, η_p_^2^ = 0.96) for TAC measurements and post hoc analysis showed that the TAC peak occurred 48 h after the match, remained significantly higher than the PRE measurement post-match and 24 h, 72 h, and 96 h after the match (*p* < 0.05), and returned almost to baseline 120 h after the match.

#### 3.7.6. Catalase

Statistical significance (F(3.57, 117.93) = 66.74, *p* < 0.001, η_p_^2^ = 0.67) was found for the CAT measurements and post hoc analysis indicated that CAT peaked immediately after the match (*p* < 0.05) and returned to baseline 24 h after the match.

#### 3.7.7. Uric Acid

Statistical significance (F(2.74, 90.40) = 348.62, *p* < 0.001, η_p_^2^ = 0.91) was found for the UA measurements and post hoc analysis revealed that UA peaked 48 h after the match, was significantly higher than the PRE measurement post-match and 24 h, 72 h, 96 h, and 120 h after the match measurements (*p* < 0.05), and returned to baseline 144 h after the match.

## 4. Discussion

**Figure 9:**
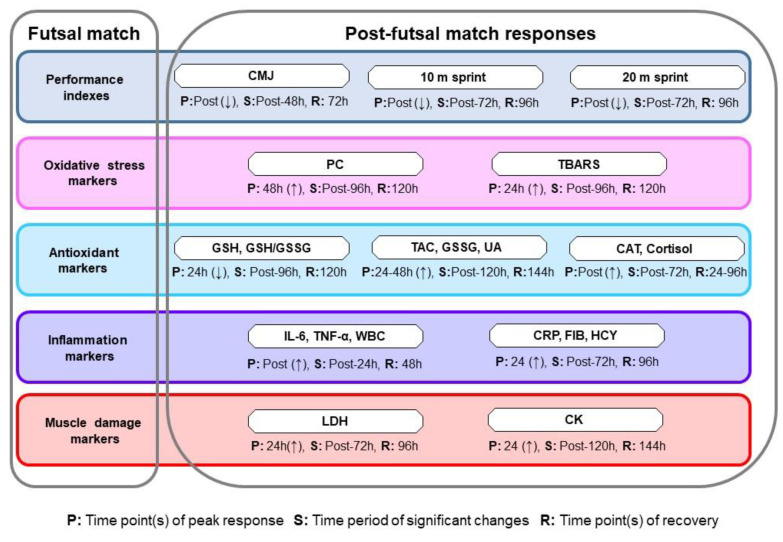
Schematic presentation of post-futsal match changes in performance indexes, oxidative stress, inflammation, and muscle damage markers. Note that an upward arrow (↑) represents an increase while a downward arrow (↓) represents a decrease in the corresponding variable(s). Abbreviations: CAT, catalase; CK, creatine kinase; CRP, C-reactive protein; FIB, fibrinogen; GSH, reduced glutathione; GSSG, oxidized glutathione; GSH/GSSG, reduced glutathione/oxidized glutathione; HCY, homocysteine; IL-6, interleukin-6; LDH: lactate dehydrogenase; TAC, total antioxidant capacity; TNF-α, tumor necrosis factor-α; UA, uric acid. WBC, white blood cell count.

The aims of this study were to investigate the time-course effects of an unofficial futsal match on performance, oxidative stress, muscle damage markers, and inflammatory and antioxidant responses during a 6-day post-match period. The findings of the study indicated that a futsal match (internal load average 85% HR_max_ and 5.5 mmol·L^−1^ blood lactate values) induced significant changes in several biochemical markers and performance parameters, i.e., decreased CMJ, 20 m, and 10 m sprints performance, and increased inflammatory, muscle damage, and oxidative stress markers for about 72 h–96 h post-match (Figure 9).

Studies in male soccer players, after a soccer match, have shown performance reduction, measured by sprint abilities and vertical jump, lasted 96 h for a 10 m sprint (4.9–9.5%), 72 h for a 20 m sprint (2.8–8.2%), and 48 h for CMJ (6.4–15.6%) [11,25]. Our study revealed similar effects of a futsal match on female athletes, which means that players may not be able to effectively perform high-intensity actions during training for a 72 h period after the match. Performance impairments during the recovery period after the match are probably associated with changes in the plasma concentration of oxidative and muscle damage markers, as well as the antioxidant capacity of the body [64]. As a straightforward, non-invasive indicator, CMJ was previously found to correlate with the GSH/GSSG ratio [65], which represents a dynamic equilibrium of oxidants and antioxidants, while its reduction is probably explained by free-radical production and the acute mobilization of the antioxidant defense system [65]. Similarly, biochemical markers such as IL-6, which acts as both a pro-inflammatory cytokine and an anti-inflammatory myokine, may promote cortisol production [66], which in turn may result in decreased physical performance due to a reduction in contractile proteins, and reduced protein synthesis, neurotransmitters, and muscular force [11,67].

The values of RPE, which is a good indicator of soccer players exertion and fatigue [68], remained high for a 72 h period after the match in the current study and for 24 h well above the observed weekly average session RPE in high-level futsal male players [69]. Moreover, as the pain and stiffness that may stem from inflammation, myofiber necrosis, and myofibrillar structure disruptions [70], DOMS values, similarly to RPE, remained elevated for extensors and flexors for a 72 h and 96 h period, respectively, in both the dominant and non-dominant legs. This phenomenon may be caused by excessive eccentric contractions of hamstrings during extension and flexion of knees and hips, at landing to the ground, while performing high-velocity movements (i.e., sprinting, tackling, jumping) [71]. Procedures such as leukocytosis or increased oxidative markers (i.e., TBARSs and UA) in the damaged tissues may explain the post-match inflammatory responses and the increased levels of subjective muscle soreness measurements [69]. A previous study showed a peak CK value 48 h after a soccer match [25], but CK and LDH biochemical markers in this study, which provide indirect evidence of muscle microtrauma, peaked 24 h after the match and remained high 72 h and 144 h after the match, respectively. This could be justified by the increased intramuscular vasculature or permeability of the plasma membrane possible due to the higher intensities of the intermittent sprints during a futsal match [18]. Consequently, regardless of the above discussion, based on our RPE and DOMS evidence, female futsal players may need at least a two-day period to recover sufficiently after a futsal match [64].

However, intense eccentric muscle activities (i.e., sprinting, jumping) result in muscle damage, which is followed by inflammatory responses [70,72]. In agreement with previous soccer [11,25,26,73,74] and futsal studies [18,75], our findings showed short/mid-term inflammatory responses. IL-6 is generally synthesized after the initial synthesis of cytokines as a result of the muscle contractions [76]. It has been found that IL-6 contributes to metabolic substrate availability and may be involved in an increase in central fatigue, overtraining, and hormonal responses to training [77]. During exercise, the IL-6 cytokine is released by muscle fibers and induces an increase in the level of other anti-inflammatory cytokines [78]. For example, CRP, which is used as a marker of chronic vascular inflammation, is synthesized in the liver in response to inflammatory factors, primarily IL-6, and to a lesser extent by TNF-α [78]. Match-induced inflammation was also verified by increased levels of WBC within damaged tissues due to post-match leukocytosis [25].

In this study, the PC concentration peaked 48 h after the match while TBARSs peaked 24 h after the match. They both remained above their normal range for a 96 h period after the match. These increases might be explained by the increased levels of catecholamines, which assist in the energy metabolism of the muscles, or the increased catabolism of purines that results in elevated levels of UA that also were observed post-match and for several days afterward [79]. Oxidative stress after a match or exercise indicates an imbalance between antioxidants and pro-oxidants due to the production of free radicals [80,81]. Moreover, the observed significant reduction in the GSH/GSSG ratio and the increase in GSSG indicate increased free-radical production and acute mobilization of the antioxidant system, which is in agreement with previous findings [65]. Regarding participants’ antioxidant status, the TAC index peaked at 48 h after the match, whereas CAT increased immediately after and returned to baseline 24 h after the match, as has been reported in a soccer study [25]. This elevated level immediately after the match probably occurred due to an increased permeability of erythrocyte membranes and CAT leakage into the circulation. On the other hand, the post-match catabolic state (i.e., increased levels of cortisol) may impair muscular force and, therefore, sprint performance [11,67].

### Limitations and Recommendations for Future Research, Strengths and Practical Implications

The generalizability of the findings of the present study is limited to adult high-level female futsal athletes playing full time (2 × 20 min) on a wooden surface. It was reasonable to assume that all participants in the experimental group had similar weekly schedules and training loads, since all of them are high-level futsal players and compete in the same top national league. On the other hand, they may have different weekly training loads and perhaps some of the participants were more fatigued at the end of the season, which may have affected the measured variables and, therefore, this constitutes another limitation of the study. However, we believe that the eleven days between the end of competitive futsal duties and the intervention match were sufficient for the players to recover from the competitive season. Τhe actual activity pattern and the distances covered in respect to the teams’ and athletes’ positions (defenders, wingers, pivots) were not recorded. However, the participants in the experimental group played almost an equal amount of time in all positions (defenders, wingers, pivots) and match intensity, as determined by the blood lactate concentration after the match [82], which was the same between the teams’ participants of the five matches played in the experimental group. Moreover, due to inconclusive results of other relative studies regarding the effect of menstruation on various performance indices, inflammation, oxidative stress, and muscle damage markers may be possibly explained by heterogeneity in monitoring and verification of menstrual cycle phase [83], our results should be compared with caution. 

Future studies are needed to evaluate the possible interaction of sex, athletes’ field position, athletes’ covered distance, and physical fitness level with performance indexes, oxidative stress, inflammation markers, and DOMS after a futsal match. Gender hormone fluctuations during the menstrual cycle may have an effect on exercise-induced muscle damage in terms of DOMS and strength loss but not on CK [84]. Muscle soreness was perceived to be more severe prior to exercise [83] and exercise performance may be marginally reduced [85] in the very early follicular phase when estrogen concentrations are low. On the contrary, female elite futsal athletes are perceived to perform better during the menstrual cycle’s follicular phase [86]. Oxidative stress during and post exercise may be eliminated in eumenorrheic females during the luteal phase, when estradiol and progesterone levels are higher than during the follicular phase [87]. Therefore, during the very early follicular phase, women’s predisposition to engage in high-intensity exercise such as a futsal match may be reduced [83]. All these combinatorically were also the main reason that the futsal match intervention was applied on day 2–8 of the follicular phase of the participants’ menstrual cycle. The percentage of potential participants in the experimental group who had irregular menstruation (8.4%) was similar to (9.6%) what has been previously reported [86], whereas the physiological characteristics of the experimental group were similar to other relevant studies [3,86], indicating the representativeness of the sample in relation to menstruation and to the physiological characteristics of female futsal players. 

At this point, it is worth noting that, to the authors’ best knowledge, this study is the first that has explored the time-course effects of a futsal match on performance indexes, oxidative stress, inflammation, and muscle damage markers, in female futsal athletes, and in six consecutive days post-match using a sound experimental design. Moreover, our outcomes give sufficient evidence to futsal coaches for the usefulness of monitoring post-futsal match residual fatigue, muscle damage, and oxidative stress markers and highlights this procedure as a valuable tool for a better weekly training workload (intensity and volume) adjustment [88] in favor of athletes’ optimum recovery. However, an individualized approach based on each player’s response to futsal performance throughout the menstrual cycle is recommended. In addition, reduced training loads or longer rest periods should be considered during the very early follicular phase, when concentrations of sex hormones are lower and female players may be more susceptible to muscle damage, while strength conditioning loads could be increased during the mid-luteal phase [84].

## 5. Conclusions

In this study, short/mid-term changes in performance indexes, inflammation, oxidative stress, and muscle damage markers for about 72 h to 96 h after a futsal match in female players are evident as in male players. In addition, the association between time course changes in several oxidative stress, inflammation, and muscle damage markers with the subjective muscle soreness, fatigue measurements and the performance indexes after a futsal match is highlighted. Although the findings of the present study derive from players who played the entire match, following a competitive match, female futsal players may also face challenges in their recovery until the next match. For that reason, and due to nature of futsal (players cover at high intensity 26% of total match distance or time, i.e., high frequency of repeated sprints < 2 s, interspersed by short recovery periods), coaches should make a large number of substitutions to mitigate the undesirable effects of the match’s high intensity, which are believed to negatively impact players’ health, physical performance, and post-match training capacity. Moreover, trainers should monitor as possible post-match residual fatigue, muscle damage, and oxidative stress, using various markers such as CRP, CK, PCs, TAC, UA, RPE, and DOMS depending on their interest, so as, based on these measurements, to adjust accordingly the weekly workload (intensity and volume) to optimize the athletes’ recovery.

## Figures and Tables

**Table 1:** Descriptive characteristics of experimental and control groups (mean ± SD [95% CI]).

Group	Experimental	Control
Age (y)	22.6 ± 2.1 [21.9–23.3]	22.3 ± 2.6 [21.4–23.3]
Height (cm)	167.4 ± 3.6 [166.1–168.6]	166.6 ± 4.6 [164.9–168.2]
Body mass (kg)	57.5 ± 3.0 [56.5–58.5]	56.8 ± 3.4 [55.6–58.0]
Body fat (%)	16.9 ± 0.8 [16.6–17.1]	16.6 ± 1 [16.2–16.9]
⩒O_2max_ (ml·kg^−1^·min^−1^)	54.6 ± 1.5 [54.1–55.2]	54.0 ± 1.4 [53.5–54.5]
Training period (y)	12.5 ± 2.3 [11.7–13.3]	12.4 ± 2.3 [11.6–13.3]
Training volume (min·week^−1^)	285.0 ± 36.3 [272.8–297.2]	274.7 ± 34.7 [262.2–287.1]
Training frequency (times·week^−1^)	5.3 ± 0.8 [5.0–5.6]	5.2 ± 0.9 [4.8–5.5]
Menarche age (y)	13.1 ± 1.0 [12.7–13.4]	13.0 ± 1.1 [12.6–13.4]
Menstrual cycle (d)	28.1 ± 1.3 [27.7–28.6]	28.4 ± 1.6 [27.8–29.0]
Intervention (d of menstrual cycle)	4.6 ± 1.8 [4.0–5.2]	4.7 ± 1.8 [4.0–5.4]

Abbreviations: SD, standard deviation; ⩒O_2max_, maximal oxygen uptake.

## Data Availability

The raw data supporting the conclusions of this article will be made available by the corresponding author upon reasonable request.
